# Construction of strains to identify novel factors for regulation of centromeric cohesion protection (CCP) and sister kinetochore mono-orientation (SKM)

**DOI:** 10.1186/s12860-019-0231-2

**Published:** 2019-10-22

**Authors:** Akhilendra Pratap Bharati, Santanu Kumar Ghosh

**Affiliations:** 1 Genomics Lab, ICAR-NBAIM, Kushmaur, Mau, India; 2 Department of Bio-sciences and Bio-engineering, IIT Bombay, Mumbai, India

**Keywords:** Centromere, cDNA library, Cohesion, Galactose, Kinetochore, Monopolin

## Abstract

**Background:**

Meiosis-I is a unique type of chromosome segregation where each chromosome aligns and segregates from its homolog. The mechanism of meiosis I homolog separation in different eukaryotes depends on their centromere and kinetochore architecture which in turn relies mainly on two processes, first on a specialized four protein complex known as monopolin and second, the centromeric cohesion protection (CCP). However, in mammals the complex has not been identified. Furthermore, in budding yeast, there could be additional factors in this process which includes some meiosis specific and some non meiosis specific factors.

**Result:**

We constructed two strains. In the first strain we expressed Mam1 and Cdc5 which leads to sister kinetochore monoorientation (SKM) and in the second case we expressed Rec8 and Spo13 which enhanced CCP even in mitosis. The expression of these proteins in mitotically dividing cells caused co-orientation of the chromosomes, which lead to the cell death followed by miss-segregation of chromosomes. Then we utilized these strains to screen the cDNA libraries from yeast and mammals to identify the novel factors which participate in CCP and SKM. Finally, SGY4119 strain expressing Spo13 and Rec8 was transformed with pRS316 gal cDNA library and transformants were screened for lethality on galactose. We screened ~ 10^5^ transformants colonies. Out of these ~ 3000 colonies were able to survive on galactose plate which was narrow down to 6 on the basis of desired phenotype.

**Conclusion:**

So far, meiosis specific kinetochore proteins have been identified only in two yeasts. Recently, in mammals a meiosis specific kinetochore protein (MEIKIN) has been identified with similar function. Till now a single protein in mammals and four proteins monopolin complex in budding yeast has been identified to coorient the centromere. Many more novel factors have to be identified yet. That is why we wished to device genetic screen using a functional genomics approach. Since the list of proteins already identified in yeast is not exhaustive as the circumstantial evidence suggests, we wish to use the same yeast strains to identify additional novel yeast proteins that may be involved in the execution of meiosis.

## Background

Transfer of the genetic information from one generation to the other through cell division is critical for survival [1]. In sexually reproducing organisms the meiotic cell division gives rise to gametes with half the chromosome number to that of the mother cell. The reduction in the chromosome number is because of the two rounds of the division without an intervening DNA replication. In the first meiotic division (MI) the homologous chromosome separates from each other. The first meiotic division is the reductional division, while the second meiotic division (MII) is equatorial division where the sister chromatids separation occurs. The homologous chromosome separation basically depends on their centromere and kinetochore architecture [2]. The kinetochore is a multiprotein complex assembles on the centromere that may span from kbs to Mbs. Each kinetochore is attached to a microtubule or multiple microtubules at the time of division [3]. The budding yeast *Saccharomyces cerevisiae* and its close relative possess ~ 150 bp centromere and can bind to a single microtubule. The homologous chromosome separation mainly depends on the mono orientation of the sister kinetochore because of which the paired homolog separates to the opposite pole. On the other side in the MII the sister kinetochore are biorient because of which the sister chromatids separates to the opposite pole. In these organisms, sister kinetochore co-orientation in MI depends mainly on two processes, first on a specialized four protein complex known as monopolin and second the CCP by Spo13, shugoshin (SGO) and protein phosphatase 2A (PP2A) [4–7].

The monopolin complex is composed of the four proteins Mam1, Csm1, Lrs4 and Hrr25. Mam1 is a meiosis specific protein, which express from the pachytene to metaphase I [8]. The Csm1 and Lrs4 express throughout the cell cycle in mitosis as well as meiosis but reside in nucleolus till G2. It is released from the nucleolus by the polo like kinase Cdc5 [9, 10]. After their release Csm1 and Lrs4 form complex with the Mam1 and bind to kinetochore [10]. Mam1 also recruits Hrr25 which also participate in the complex formation [11]. It means that if we express Cdc5 and Mam1 in mitotically dividing cell it can form the monopolin complex. The Csm1 and Lrs4 form a V-shaped complex with two globular “heads” spaced ~ 10 nm apart. Each head comprises a dimer of Csm1 C-terminal domains, and each of these domains can bind the kinetochore proteins Dsn1 and Mif2. Thus, the full complex has two pairs of kinetochore binding sites separated by ~ 10 nm [12, 13]. Similarly, in *S. pombe* the Pcs1 form a complex with Mde4 and both localizes to the core of the centromere like monopolin complex. These two proteins don’t participate in the monoorientation of sister kinetochore but play an important role in the amphitelic kinetochore orientation during MII and in mitotic division [14].

The centromeric cohesin during MI is mainly replaced by Rec8. At the time of anaphase I the Rec8 is cleaved by seperase along the chromosome arm region, but protected in the centromeric region [15, 16]. The destruction of the cohesion along the arm resolves the chiasmata and this event produce the univalent chromosomes, which are held together by the centromeric cohesion [15]. The cohesion protection in the centromeric region is mediated by the Shugoshin which recruit the phosphatase PP2A at the centromere [15, 16]. In the presence of PP2A the Rec8 remains unphosphorylated which is resistant to cleavage by separase [5, 6]. Shugoshin was identified by several groups in 2004 having a role in the CCP in different organisms [17–19]. Marstan et al., 2004 collected the *Saccharomyces cerevisiae* strains with individual genes deleted and having GFP dots on both the copy of Chromosome III. They observed the non-disjunction and premature separation of the chromosomes in some strains. A similar observation was done in fission yeast where Shugosin was recruited to the centromere by Bub1 [17] and same homologs were identified in the Drosophila as well at the same time [19].

Spo13 has also been reported to play role in centromeric cohesion protection [4, 20]. Despite the genome-wide association of Spo13 it protects only the centromeric cohesion in MI, the mechanism is still unknown [7]. Till now, Spo13 and Mam1 in *Saccharomyces cerevisiae* and Moa1 in *S. pombe* is the only meiosis specific kinetochore protein identified. The meiosis-specific protein Spo13 is also necessary for kinetochore co-orientation. In its absence, the monopolin complex initially associates with kinetochore but cannot be maintained there [4, 7, 21]. How the monopolin complex and proteins that regulate its association with kinetochore bring about sister kinetochore co-orientation is poorly understood. These examples suggest that the existing list of kinetochore proteins is not sufficient and it needs to be updated. Beside the kinetochore protein, the formation of the chiasmata also play crucial role in the attachment of the sister chromatids to the same spindle pole [22, 23]. SKM and CCP in addition to the formation of chiasmata ensure the reductional nature of the chromosome segregation in MI [22]. Moreover, the modification of the kinetochore protein also plays role in the SKM. Acetylation of the Psm3 by Eso1 is required for the monoorientation in fission yeast [24]. Thus, SKM and CCP are two hallmarks of meiotic kinetochore function, which are widely conserved among eukaryotic organisms. However, the structural and functional similarities remain to be identified and even conservation of meiotic kinetochore regulation is questionable even between yeasts. Beside this a number of missing links are there which suggest that there may be the possibility of other meiosis and non-meiosis factors which also participate in this phenomenon [25].

To find out the additional proteins responsible for the SKM and CCP, we started with the construction of two strains. In first strain we expressed Mam1 and Cdc5 to attain the forceful kinetochore co-orientation in mitosis. Cdc5 expression helps the Lrs4 and Csm1 to localize in the nucleus so that it can form the monopolin complex with Mam1 even in mitotic cell division. In the second strain we are expressing the Rec8 and Spo13 to increase the centromeric cohesion protection. So in these strains we are forcefully generating the meiosis I condition. In case of mitotic division the cells were found to be sick, utilizing this phenotype, we can screen the specific cDNA library to find out the novel factors. This report shows the simple methods to screen the novel factors from the cDNA library for a specific pathway or process.

## Results and discussion

### Construction of engineered strains showing ‘sick’ phenotype

**Fig. 1:**
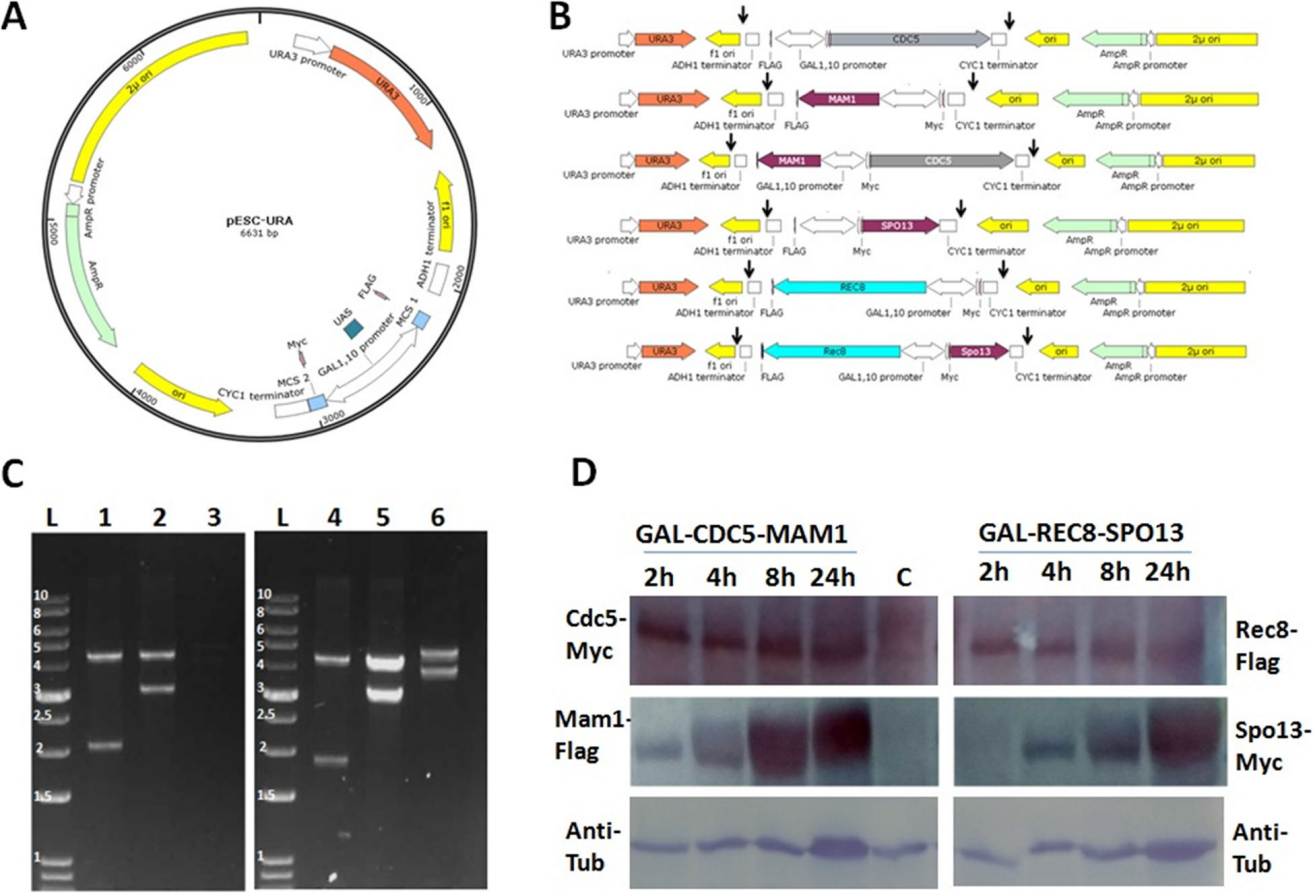
Construction of engineered strains. **a** Diagrammatic representation of pESC-URA vector. **b** Construct cloned in pESC-URA along with the gal promoter and corresponding terminator. The black arrow represents the site which was amplified by PCR for the cloning in the pRS406 integration vector. **c** Restriction digestion of pRS406 constructs using *Hind*III and *Not*I. L stands for ladder, lane 1 represent the digestion of pRS406-GAL-MAM1 construct similarly 2, 3, 4, 5 and 6 represent the pRS406-GAL-CDC5, pRS406-GAL-CDC5-MAM1, pRS406-GAL-SPO13, pRS406-GAL-REC8, pRS406-GAL-SPO13-REC8 respectively. **d** CRY1 strain was transformed with pESC-GAL-CDC5-MAM1-URA (GAL-Cdc5-Mam1), pESC-GAL-SPO13-REC8-URA (GAL-Rec8-Spo13) and pESC-URA (c) construct and single colony was picked and checked for the expression of these proteins at different time point after galactose induction as described in methodology

*MAM1* and *CDC5* genes from *S. cerevisiae* was cloned into pESC-URA to form pESC-CDC5-MAM1-URA construct, similarly the pESC-REC8-SPO13-URA construct was made as described in the methodology (Fig. 1a, b). pESC-URA is a 2 μ high copy number plasmid. To restrict the single copy per cell, we again amplified these expression cassette from pESC-URA construct using APB008 and APB009 primer (the amplified portion is marked with a black head arrow in Fig. 1c) and cloned in integration vector (pRS405) between *Hind*III and *Not*I site. Now these integration constructs were linearized by *Stu*I restriction enzyme and integrated in CRY1. The expressions of the cloned genes were checked after galactose induction at different time interval (Fig. 1d). The result indicated that the expression of the protein was increased with an increase in time. We checked the expression of tubulin in each sample as a control. The growth of these two strains was checked and found to be very slow growing in synthetic complete media supplemented with 2% galactose as compared to 2% dextrose (Fig. 3a, lane 4 and Fig. 3b, lane 4). It means that the expression of the protein was capable to restrict the division of the cells or affect the viability as a result the cells were sick.

### Chromosomal segregation is compromised in engineered strains

**Fig. 2:**
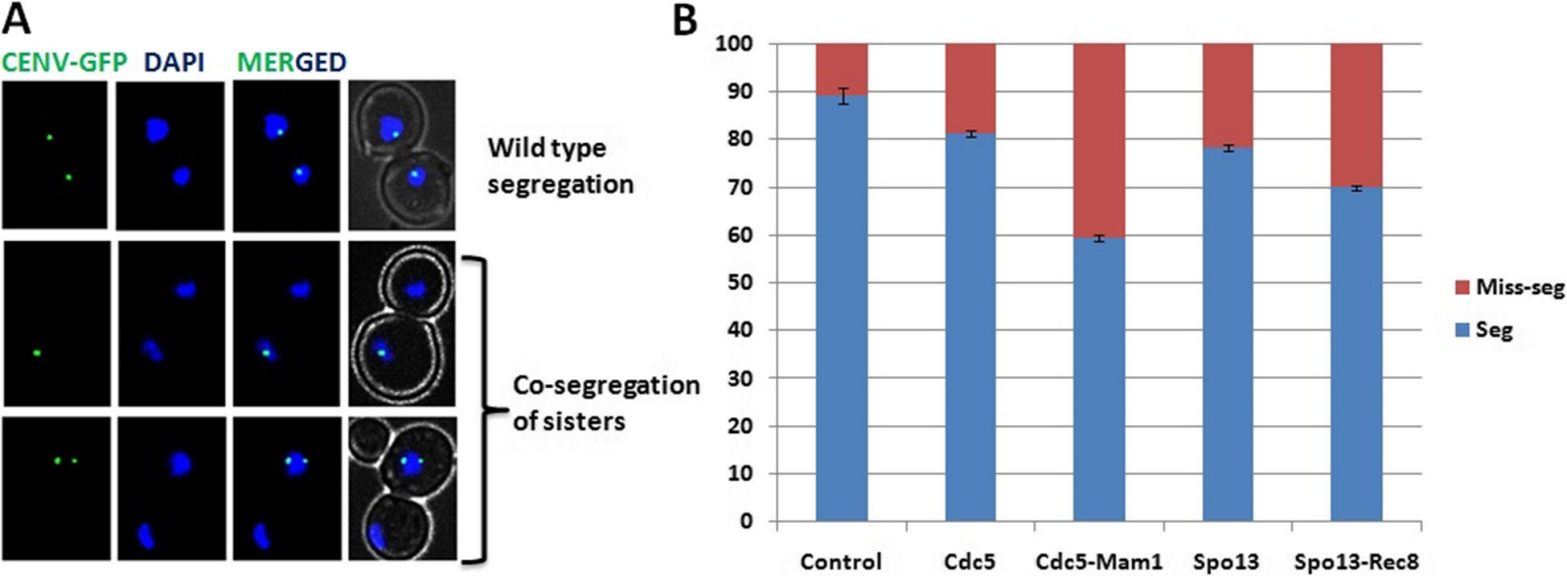
Mitotic chromosomal segregation is compromised in engineered strains. **a** Wild type (SGY229) as well as transformed strains were grown till 0.8 OD_600_ and then induced with 2% galactose for 4 h. Samples were collected and fixed with formaldehyde and live cell imaging was done to see the CEN-V GFP segregation. **b** For each strain, control (SGY229 integrated with pRS405-URA), Mam1 (SGY229 integrated with pRS405-MAM1), Cdc5 (SGY229 integrated with pRS405-CDC5), Cdc5Mam1 (SGY229 integrated with pRS405-CDC5-MAM1) and Spo13 (SGY229 integrated with pRS405-SPO13) the cell for wild type segregation and co-segregation was counted and plotted. For statistical calculation this experiment was done in triplicate and more than 100 cells were counted in each case to see the segregation verses miss-segregation

To find out the effect of the over-expression of these proteins (Rec8, Spo13, Mam1 and Cdc5) on chromosomal segregation we first integrated a tandem array of tetO sequences near the centromere of chromosome V (SGY229). These cells also expressed a tetR-GFP fusion, which binds to tetO, to visualize the repeats. We transformed this strain with the *Stu*I digested pRS406 construct along with the cloned genes (pRS406, pRS406-CDC5, pRS406-MAM1, pRS406-CDC5-MAM1, pRS406-REC8, pRS406-SPO13, and pRS406-REC8-MAM1 briefly notified as control, Cdc5, Mam1, Cdc5-Mam1, Rec8, Spo13, Rec8-Spo13) and the transformants were grown in SC-URA broth supplemented with 2% raffinose till 0.8 OD_600_. These samples were induced with the 2% of galactose till 4 h and the samples were prepared for the DAPI staining and the cells were observed under fluorescence microscopy. We observed two types of cells, first was properly segregated having GFP dot with proper DAPI segregated (Fig. 2a, wild type segregation) and second miss-segregated cells where DAPI was segregated, but either one of the cell was not having GFP dot and another was having two or one dot (Fig. 2a, co-segregation of sisters). The microscopy results showed that alone Mam1 or Rec8 expression had not much effect on segregation (data not shown) while expression of Spo13 or Cdc5 alone led to ~ 20% miss-segregation and expression of both Cdc5-Mam1 and Rec8-Spo13 did lead to more than 40 and 30% miss-segregation respectively (Fig. 2b). These results also satisfied the previous reports of miss-segregation of chromosomes [20, 26]. It means our strains were working fine at the chromosomal segregation level. So at the molecular level the strains can be utilized for the screening of the novel factors responsible for the chromosomal segregation from the cDNA library.

### Mitotic expression of Spo13 or Cdc5 is detrimental to cell growth

**Fig. 3:**
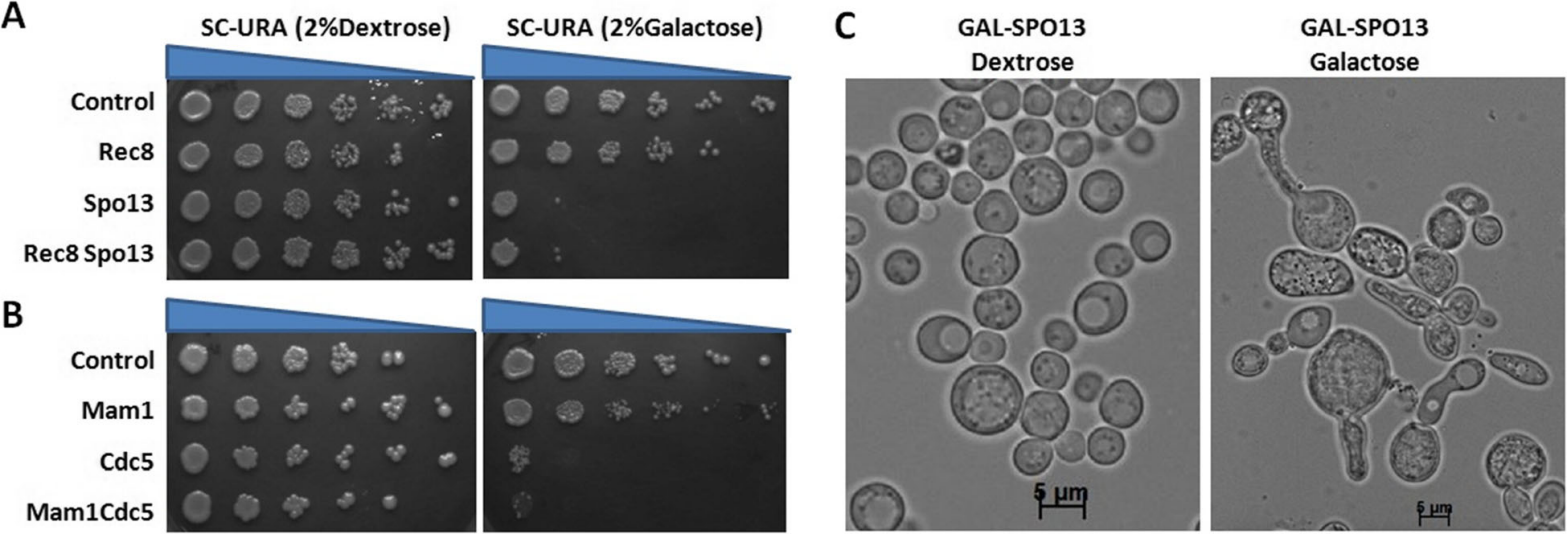
Spo13 and Cdc5 overexpression affect the viability of cells. **a** Frogging assay of CRY1 integrated with pRS405-URA (SGY4104), pRS405-REC8 (SGY4108), pRS405-SPO13 (SGY4103) and pRS405-REC8-SPO13 (SGY4110) respectively (control, Rec8, Spo13, Rec8Spo13) were first inoculated at 30 °C for overnight in 5 ml SC-uracil broth supplemented with raffinose, now the OD_600_ of the broth was checked, make the OD_600_ of each broth ~ 1 by dilution using same broth, then make the dilution and spot them on the SC-URA supplemented with galactose (right panel) or dextrose (left panel) as a carbon source. **b** Frogging assay of CRY1 integrated with pRS405-URA (SGY4104), pRS405-MAM1 (SGY4106), pRS405-CDC5 (SGY4105) and pRS405-MAM1-CDC5 (SGY4107) respectively (control, Mam1, Cdc5, Mam1Cdc5) on SC-uracil plate supplemented with galactose (right panel) or dextrose (left panel) similarly as mentioned earlier. **c** Cell morphology of SGY4112 (GAL-SPO13) strain in dextrose and galactose

To check the effect of the expression of Rec8 and Spo13 on growth of the cells, the construct were integrated in CRY1 and spotting was done on galactose as well as on dextrose plate (as described in method). We observed that the growth on the dextrose plate (Fig. 3a, b, left side) was normal while there was a defect on the galactose plate in some strains (Fig. 3a and b). In Fig. 3a, right panel, second row the strain SGY4108 (integrated with the pRS406-REC8 construct) was growing normal on galactose. It means the expression of Rec8 alone had no growth defect. On the other hand the growth of the strains SGY4109 and SGY4110 (integrated with pRS406-SPO13 and pRS406-REC8-SPO13 construct respectively) had a detrimental effect on the growth. It means the Spo13 expression alone was toxic to the cell, but the combination of both had an additional detrimental effect on the chromosomal segregation as Fig. 2b had already suggested. It means that the effect of Spo13 expression was more lethal as compared to Rec8 expression. Varela et al. first reported that mitotic Spo13 expression altered cell cycle progression and was unable to exit anaphase with change in the cell morphology [27]. We also observed same morphological change (elongated cell) in Spo13 expression strain (Fig. 3c, right side). It means slower growth on galactose was not because of the enhanced CCP only, it might be because of Spo13 expression also. This might be the reason of getting the same growth defect in Spo13 as well as Spo13-Rec8 (Fig. 3a, upper panel).

Similarly, we checked the effect of Mam1 and Cdc5 expression on the growth of the strains (Fig. 3b). Strain SGY4106 (integrated with pRS406-MAM1) was growing normal on galactose while strain SGY4105 and SGY4107 (integrated with pRS406-CDC5 and pRS406-CDC5-MAM1 respectively) was growing at the same rate and was very slow growing as compare to SGY4106 strain (Fig. 3b). It means the Cdc5 expression alone could restrict the cell growth. But in the Fig. 2a the effect of Cdc5 and Mam1 was almost equal on chromosomal segregation. Cdc5 expression led to the metaphase arrest, which maybe because of the increased APC activity [28]. This might be the reason of getting same growth defect in both Cdc5 and Cdc5-Mam1 expression (Fig. 3a, lower panel). If this defect was because of the increased APC activity, then we could rule out this using the APC mutant strain (*cdc16–1, cdc16–123, cdc20–1*) which had compromised APC activity.

### Growth defect because of Cdc5 over-expression is rescued in *Cdc16–1* strain

**Fig. 4:**
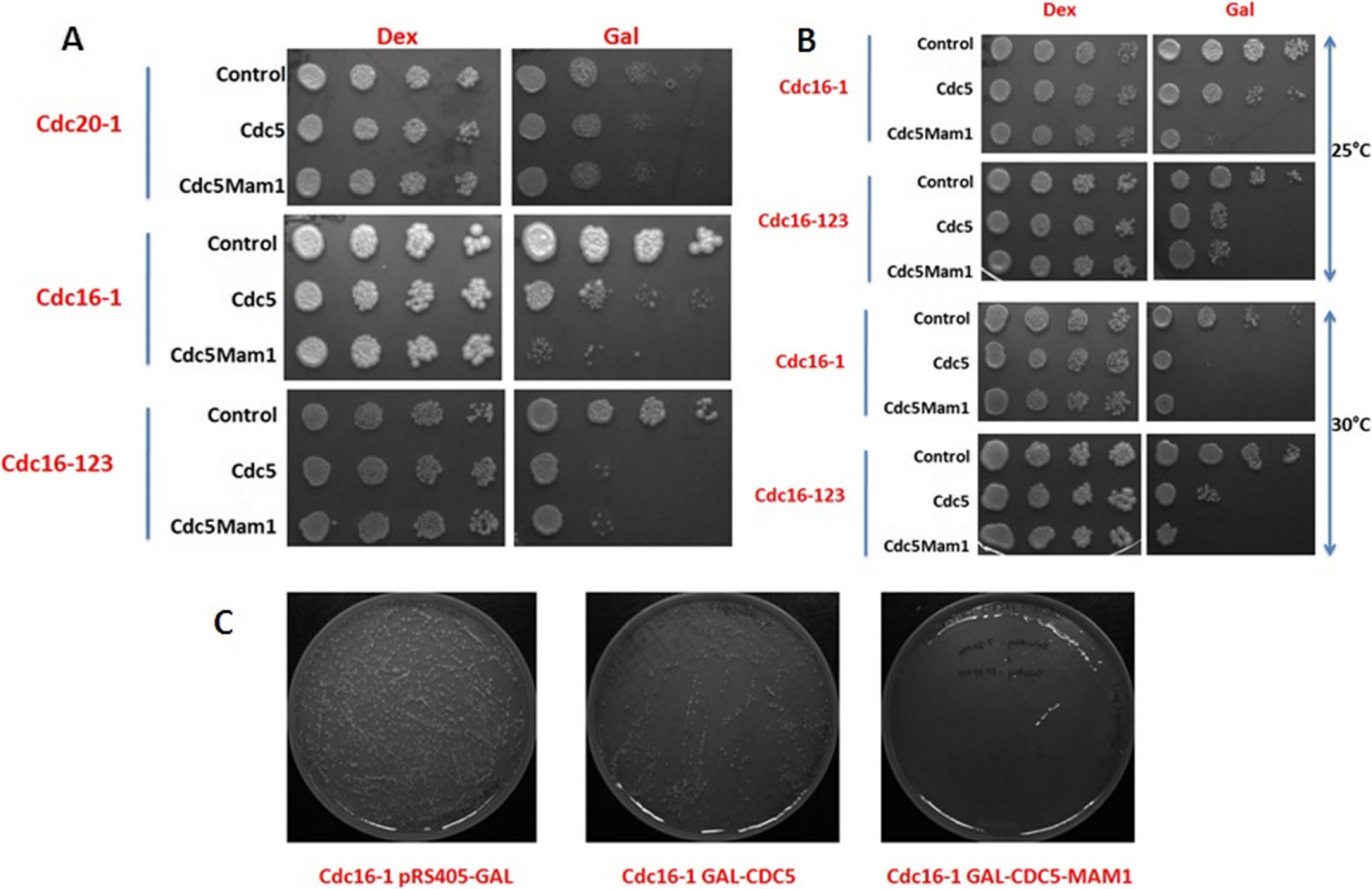
Growth defect because of Cdc5 over-expression is rescued in *cdc16–1* strain. **a** APC mutant strains *cdc16–123*, *cdc16–1*, *cdc20–1* were integrated with pRS405-Leu, pRS405-CDC5-Leu and using pRS405-CDC5-MAM1-Leu independently to make SGY4129, SGY4130, SGY4131, SGY4132, SGY4133, SGY4134, SGY4135, SGY4136 and SGY4137 strains. These strains were grown and frogging was done using equal amount of cell at 23 °C. **b** *cdc16–123*, *cdc16–1* integrated with pRS405-Leu, pRS405-CDC5-Leu and using pRS405-CDC5-MAM1-Leu independently (SGY4129, SGY4130, SGY4131, SGY4132, SGY4133 and SGY4134 strains) were grown and frogging was done at two different temperature (25 °C, 30 °C). **c** SGY4132, SGY4133 and SGY4134 were transformed with pRS316-gal empty vector and equal volume of the transformats was spread on SC-leu-ura plate supplemented with galactose and incubated at 30 °C

Furthermore, we thought that the over expression of the Cdc5 leads to the death of the cell because of the increased APC activity as Cdc5 expression enhances excessive APC activity [28]. We thought that the use of the APC mutants may serve our purpose. So we used three APC mutants to test our hypothesis (*cdc16–123*, *cdc16–1*, *cdc20–1*). We first cloned *CDC5* and *CDC5-MAM1* along with the gal promoter in the pRS405-LEU vector. We integrated pRS405, Cdc5 (using pRS405-CDC5-Leu) and Cdc5-Mam1 (using pRS405-CDC5-MAM1-Leu) independently along with the gal promoter in *cdc16–123*, *cdc16–1*, *cdc20–1* to make SGY4129, SGY4130, SGY4131, SGY4132, SGY4133, SGY4134, SGY4135, SGY4136 and SGY4137 strains respectively. The transformants were checked for the expression of Cdc5 and Mam1 in these strains (data not shown). Then we did the frogging on galactose as well as on dextrose plates at 23 °C (Fig. 4a, upper panel). We found that the growth of SGY4135, SGY4136 and SGY4137 strains (*cdc20–1* transformed with pRS405-leu, Cdc5 and Cdc5-Mam1) were very slow at 23 °C (Fig. 3a) and were unable to grow at 25 °C and 30 °C (data not shown) beside this, there was no such difference in the growth pattern at 23 °C on galactose plate in between SGY4135, SGY4136 and SGY4137 strains (Fig. 4a, upper panel). *Cdc16–1* transformed with pRS405-leu, pRS405-CDC5-Leu, pRS405-CDC5-MAM1-Leu (SGY4132, SGY4133 and SGY4134 strains, respectively) and *cdc16–123* transformed with pRS405-leu, pRS405-CDC5-Leu, pRS405-CDC5-MAM1-Leu (SGY4129, SGY4130 and SGY4131 strains, respectively) grew normally at 23 °C, 25 °C, 30 °C (Fig. 4a, middle and lower panel, Fig. 4b). We checked the growth of SGY4129, SGY4130 and SGY4131 strains at all the three temperatures, but found no difference in the growth (Fig. 4a last row, Fig. 4b second and fourth row). We found that SGY4133 strain (*cdc16–1* transformed with pRS405-CDC5-Leu) was slow growing as compared to SGY4132 (*cdc16–1* transformed with pRS405-Leu), but SGY4134 strain (*cdc16–1* transformed with pRS405-CDC5-MAM1-Leu) was unable to grow (Fig. 4a middle panel, Fig. 4b first and third panel). We also checked the growth of these strains on 37 °C, but the strains were unable to grow. Finally, we concluded that SGY4134 strain with *cdc16–1* mutant was appropriate for the library screening, where SGY4133 expressing with the Cdc5 was able to grow slowly, but SGY4134 expressing with Cdc5 along with Mam1 was not viable. Furthermore, to confirm this result, we took an equal number of cells (equal OD_600_) of SGY4132, SGY4133 and SGY4134 strains respectively and were transformed with pRS316-gal empty vector. In every transformation equal amount of chemical was used and lastly equal volume of cells was spread from SGY4132, SGY4133 and SGY4134 strains on SC-leu-ura plate supplemented with galactose and found no colony in strain SGY4134 (Fig. 4c, third plate), while there was a little number of colonies in strain SGY4133 (Fig. 4c, second plate) as compare to control strain SGY4132 (Fig. 4c, first plate). It means in *cdc16–1* the Cdc5 alone was not responsible for the growth defect when it came with the Mam1 the effect is lethal. Finally, we concluded that *cdc16–1* mutant is appropriate for the library screening.

### Library screening against SGY4119 strain

**Fig. 5:**
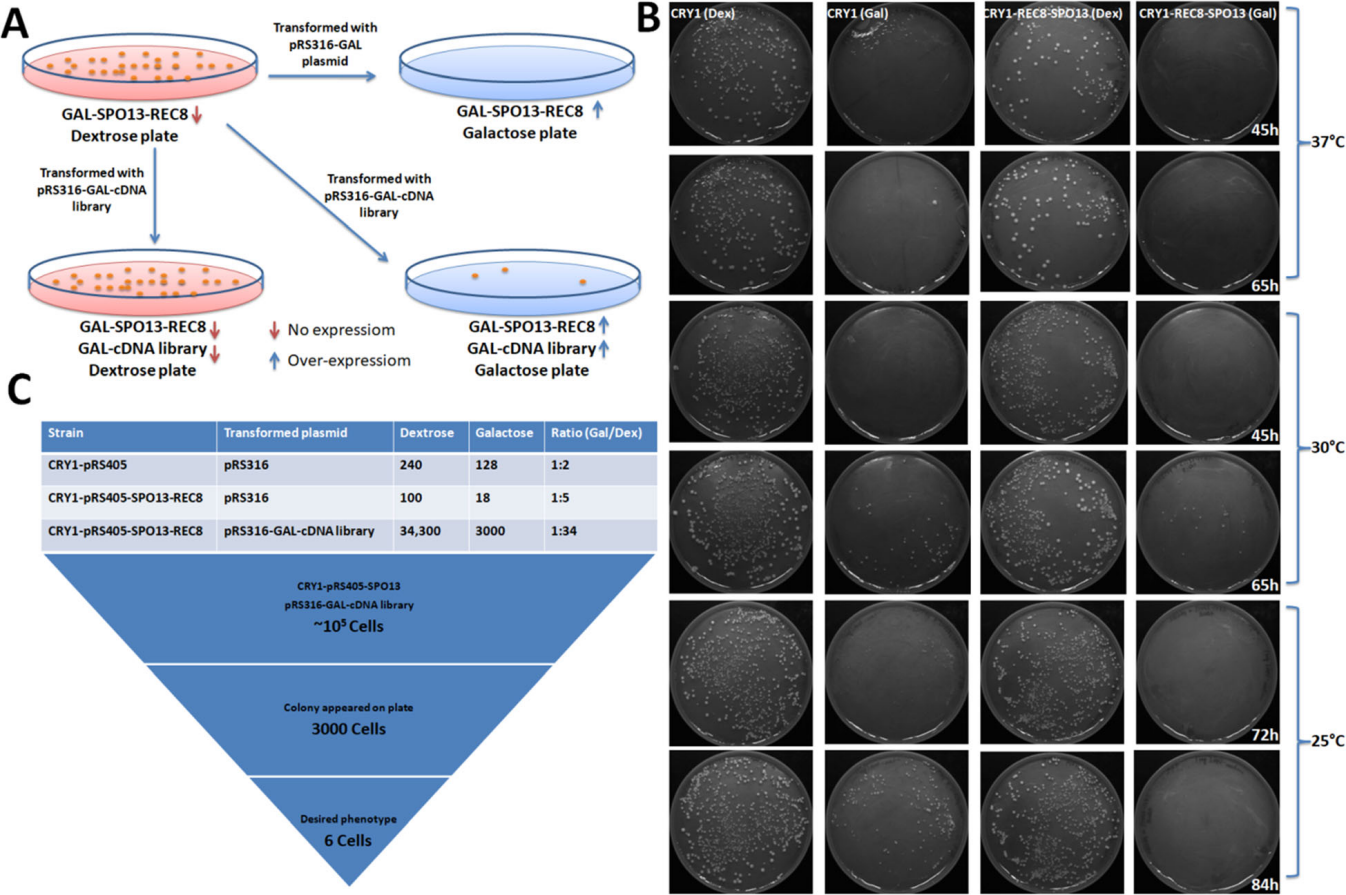
Library screening against SGY4119 strain. **a** Diagrammatic representation of the hypothesis used for the screening. Left side plates are dextrose plates while that of the right side is galactose plate. Red arrow headed downside represents no expression of that gene, while the blue arrow headed upside represents the overexpression of the proteins. **b** SGY4111 (CRY1 transformed with pRS405) and SGY4119 was transformed with the pRS316-Gal empty vector and plated on galactose and dextrose and incubated at three different temperature 37 °C, 30 °C and 25 °C and picture was taken at different time point. **c** SGY4111 was transformed with pRS316-gal and SGY4119 was transformed with pRS316-gal and pRS316-gal-cDNA library separately. In all the three cases equal number of cells were spread on galactose and dextrose plates. Plates were incubated on 30 °C and colony appeared on the plates were counted ratio of viable and non-viable were counted. SGY4119 strain transformed with the pRS316-gal-cDNA library and ~ 10^5^ cells were spread on galactose but only ~ 3000 cells appeared

The hypothesis of screening is explained in the Fig. 5a. The strain SGY4119 grow normally on a dextrose plate (Fig. 5a, left side, upper plates) while galctose plate allows very few cells to grow because of the Spo13 and Rec8 over-expression. The SGY4119 strain transformed with the library (pRS316-gal-cDNA library) and was spread on SC-leu-ura plate supplemented with the galactose leads to the further reduction in the number of colonies (Fig. 5a right side, upper plate). The transformants which were not able to survive on galactose because of the overexpression of Spo13 and Rec8, the effect may overcome because of some factors which might involved in the same pathway and can rescued the detrimental effect of Spo13 and Rec8 (Fig. 5a right side, lower plate). These factors might have a role in the segregation of the chromosomes along with the Spo13 and Rec8. On the other hand the overexpression of additional factor might lead to the death of the colonies also. So there are two factors, first is the galactose which itself limit some of the colonies to grow because of the fitness factor and the second is the gene of the library transformed which get over-expressed on the galactose plate which further act as the second factor (in combination with Spo13 and Rec8) leads to death of the colonies.

First of all we tried to standardize the procedure for the screening where control strain SGY4111 (CRY1 integrated with pRS405) and SGY4119 was transformed with empty vector pRS316-GAL (used to prepare library). After transformation both the samples were spread on SC-leu-ura plate supplemented with either galactose or dextrose at three different temperatures 25 °C, 30 °C, 37 °C simultaneously. Plates were observed on the regular interval. We observed that the appearance of the colonies on galactose plate was delayed as compared to the dextrose plate. The transformants of the dextrose plate grew normally while transformants on galactose plate appeared after 45 h of incubation which get prominent after 65 h at 30 °C (Fig. 5b, middle two panels). At 37 °C the colonies were not able to grow on galactose plate even after 65 h (Fig. 5b, upper two panels). We also observed that the growth on 23 °C was very slow as compared to 30 °C as it took 72 h to appear the colony and 84 h to get it prominent. It means that 30 °C temperature was optimal for screening and we could wait for 45 h only to get the prominent transformants for the screening.

We checked the efficiency of the pRS316-gal-cDNA library [29] by digesting 15 clones using *Sal*I and *Not*I restriction enzyme (Additional file 1: Figure S1A). These restriction sites were utilized in cDNA preparation. We got 14 out of 15 clones along with inserts. It means that the 93% of the clones are having c-DNA insert with an average insert size of 873 bp. On the basis of this information, we estimated that the frequency of a single gene existing in the c-DNA library is about one in ~ 14,900 clones (Additional file 1: Figure S1A). On the basis of this information we screened the c-DNA library. We also checked the transformation efficiency of the library by transforming 850 ng of the cDNA library into the yeast strain (CRY1) which was found to be ~ 3 × 10^5^ (Additional file 1: Figure S1B). It means only 26 ng of cDNA was required to get 15,000 transformants.

SGY4119 was transformed with the cDNA library along with some controls (SGY4111 and SGY4119 transformed with pRS316-GAL empty vector). The equal volume of the transformed cells was spread in each experiment so that the number of cells remains constant on each plate. The transformants were selected on SC-leu-ura medium supplemented with galactose or dextrose and colonies were counted. We found that the survivability on the galactose plate of SGY4119 was very less as compared to controls (Fig. 5c). In case of control where SGY4119 was transformed with pRS316-GAL empty vector, one cell survived on galactose out of five (1:5), whereas in case of cDNA library transformation one cell survived out of 34 (1:34) on galactose if we compared it with that of dextrose (for comparison we plated equal number of cells on both galactose as well as dextrose plate). Here we screen ~ 10^5^ colonies, which represent ~ 6.7 times of the required colonies as the frequency of a single copy mRNA existing in the cDNA library is about 1 in 14,900 colonies. Out of ~ 10^5^ cells ~ 3000 appeared on galactose plate.

Further, we analyzed that there were two types of colonies, in the first type of colony, the phenotype was flat and color was little whitish and look like a sick colony. We checked the cell under microscope and found that the cells were elongated as we mentioned earlier (Fig. 3c, right side). In the second type of colony the phenotype was domed shaped, little pale and cell looked healthier (Fig. 3c, left side). In the microscope the cells looked normal. During the screening, we found only six colonies of second type rest were of the first type. We isolated those six colonies and purified them, after that the plasmid carrying the cDNA were isolated. We first checked the plasmid by digesting with *Sal*I and *Cla*I and send for the sequencing to identify the gene cloned in the vector. This is the simple method to identify novel factors in any organism using cDNA library. Similarly, we can use the SGY4134 to identify the additional factors working in sister kinetochore mono orientation.

## Conclusion

So far, meiosis specific kinetochore proteins have been identified only in two yeasts. However, their structural and functional similarities remain to be identified and conservation of meiotic kinetochore regulation is questionable even between yeasts. In the absence of the monopolin complex, sister chromatids bi-orient in meiosis I, but fail to segregate since cohesin in the centromeric region is preserved. So, there may be some other factors which are also responsible to maintain the cohesion. Recently, in mammals a meiosis specific kinetochore protein (MEIKIN) has been identified with similar function [30]. But, the whole monopolin complex has to be identified in case of mammals. Since the list of proteins already identified in yeast is not exhaustive as the circumstantial evidence suggests, we wish to use the same yeast strains to also identify additional novel proteins that may be involved in the execution of meiosis in different organisms.

## Method

### Yeast strains

For gene integration, promoter shuffling, C-terminal and N-terminal tagging of proteins, PCR-based approach was adopted using appropriate plasmid borne cassettes obtained from Euroscarf. C-terminal and N-terminal protein fusions were verified both by PCR and Western blotting (all primers are listed in the Table 1). All the strains used in this study (listed in Table 2) were isogenic to CRY1 background. Marking of SGY229 was already described in Mehta et al., 2014 [31]. APC mutant strains like *cdc16–1*, *cdc20–1* and *cdc20–123* were a kind gift from the Prof. M. Jayaram, University of Taxus. *cdc16–1*, *cdc20–1* and *cdc20–123* strains were modified by integration.

**Table 1:** List of primers used in this study

Primer name	Sequence	Remark
GM105	atgcatgcatgcatgcctcgagatggcacccagaaaacgc	Forward primer with *Xho*I restriction site for SPO13 amplification
GM106	atgcatgcatgcatgcgctagcttaattaagggaagactcactatc	Reverse primer with *Nhe*I restriction site for SPO13 amplification
MA113	atgcatgcatgcatgcgaattcatggcacctctttcgttg	Forward primer with *EcoR*I restriction site for REC8 amplification
APB004	ggactagtaaggcatatacaattatttcg	Reverse primer with *Spe*I restriction site for REC8 amplification
MA111	atgcatgcatgcatgcgaattcatgagggaaaaaagaacaat	Forward primer with *EcoR*I restriction site for MAM1 amplification
APB003	ggactagtaaattttcatctatatgtagcttt	Reverse primer with *Spe*I restriction site for MAM1 amplification
MA72	atgcatgcatgcatgcctcgagatgtcgttgggtcctcttaa	Forward primer with *Xho*I restriction site for CDC5 amplification
MA73	atgcatgcatgcatgcgctagcttaatctacggtaacaat	Reverse primer with *Nhe*I restriction site for CDC5 amplification
APB008	ataagaatgcggccgcaactgttgggaagggcgatc	Forward primer with *Not*I restriction site anneal at ADH1 terminator
APB009	cccaagcttatacgcaaaccgcctctccccgc	Reverse primer with *Hind*III restriction site anneal at CYC1 terminator

**Table 2:** List of the strains prepared and used in this study

Strain ID	Nick Name	Genotype	Background
SGY229	WT CENV-GFP	Mat-a, leu2::tetR-GFP::LEU2::TetO-HIS3	CRY1
SGY4104	CRY1-pRS406-control	Mat-a ade2–1 ura3–1 leu2–3112,trp1,his3–11::GAL -URA	CRY1
SGY4105	CRY1-pRS406-GAL-CDC5	Mat-a ade2–1 ura3–1 leu2–3112,trp1,his3–11::GAL -CDC5-URA	CRY1
SGY4106	CRY1-pRS406-GAL-MAM1	Mat-a ade2–1 ura3–1 leu2–3112,trp1,his3–11::GAL - MAM1-URA	CRY1
SGY4107	CRY1-pRS406-GAL-CDC4-MAM1	Mat-a ade2–1 ura3–1 leu2–3112,trp1,his3–11::GAL - CDC4-MAM1-URA	CRY1
SGY4108	CRY1-pRS406-GAL-REC8	Mat-a ade2–1 ura3–1 leu2–3112,trp1,his3–11::GAL - REC8-URA	CRY1
SGY4109	CRY1-pRS406-GAL-SPO13	Mat-a ade2–1 ura3–1 leu2–3112,trp1,his3–11::GAL - SPO13-URA	CRY1
SGY4110	CRY1-pRS406-GAL-REC8-SPO13	Mat-a ade2–1 ura3–1 leu2–3112,trp1,his3–11::GAL - REC8-SPO13-URA	CRY1
SGY4111	CRY1-pRS405	Mat-a ade2–1 ura3–1 leu2–3112,trp1,his3–11::GAL-LEU	CRY1
SGY4112	CRY1-pRS405-GAL-SPO13	Mat-a ade2–1 ura3–1 leu2–3112,trp1,his3–11::GAL-SPO13-LEU	CRY1
SGY4113	CRY1-pRS405-GAL-CDC5	Mat-a ade2–1 ura3–1 leu2–3112,trp1,his3–11::GAL-CDC5-LEU	CRY1
SGY4114	CRY1-pRS405-GAL-CDC5-MAM1	Mat-a ade2–1 ura3–1 leu2–3112,trp1,his3–11::GAL-CDC5-MAM1-LEU	CRY1
SGY4119	CRY1-pRS405-GAL-SPO13-REC8	Mat-a ade2–1 ura3–1 leu2–3112,trp1,his3–11::GAL-SPO13-REC8-LEU	CRY1
SGY4129	Cdc16–123-pRS405	Mat-a ade2–1 ura3–1 leu2–3112,trp1,his3–11::GAL-LEU	Cdc16–1
SGY4130	Cdc16–123-pRS405-CDC5	Mat-a ade2–1 ura3–1 leu2–3112,trp1,his3–11::GAL-CDC5-LEU	Cdc16–1
SGY4131	Cdc16–123-pRS405-CDC5-MAM1	Mat-a ade2–1 ura3–1 leu2–3112,trp1,his3–11::GAL-CDC5-MAM1-LEU	Cdc16–1
SGY4132	Cdc16–1-pRS405	Mat-a ade2–1 ura3–1 leu2–3112,trp1,his3–11::GAL-LEU	Cdc16–123
SGY4133	Cdc16–1-pRS405-CDC5	Mat-a ade2–1 ura3–1 leu2–3112,trp1,his3–11::GAL-CDC5-LEU	Cdc16–123
SGY4134	Cdc16–1-pRS405-CDC5-MAM1	Mat-a ade2–1 ura3–1 leu2–3112,trp1,his3–11::GAL-CDC5-MAM1-LEU	Cdc16–123
SGY4135	Cdc20–1-pRS405	Mat-a ade2–1 ura3–1 leu2–3112,trp1,his3–11::GAL-LEU	Cdc20–1
SGY4136	Cdc20–1-pRS405-CDC5	Mat-a ade2–1 ura3–1 leu2–3112,trp1,his3–11::GAL-CDC5-LEU	Cdc20–1
SGY4137	Cdc20–1-pRS405-CDC5-MAM1	Mat-a ade2–1 ura3–1 leu2–3112,trp1,his3–11::GAL-CDC5-MAM1-LEU	Cdc20–1

### Cloning of the construct and their expression in yeast

*MAM1* and *CDC5* were cloned in pESC-URA (Fig. 1a) vector to form pESC-CDC5-MAM1-URA construct. First *CDC5* was cloned between *Xho*I and *Nhe*I site so that it was in frame with N-terminal myc tag downstream of the GAL promoter. Then in the same construct *MAM1* was cloned between the *EcoR*I and *Spe*I in such way that it should be in frame with c-terminal flag tag. Similarly the pESC-REC8-SPO13-URA construct was made by cloning *REC8* in between *Xho*I/*Nhe*I and *SPO13* in between the *EcoR*I/*Spe*I. Wild type haploid strains (CRY1) were transformed with these construct and expression of the cloned proteins was checked after galactose induction, we first grow the cells in SC broth lacking uracil for overnight in 5 ml tube (30 °C, 200 rpm shaking). Secondary inoculation was done in 50 ml flask and initial OD_600_ was set 0.2 and grown in same condition. The cells were grown till 0.8 OD_600_, induction was done with 2% galactose in same condition. Protein extraction was done by NaOH method [32]. Protein was transferred to PVDF membrane followed by SDS-PAGE. To check the expression of myc, flag tagged proteins and tubulin western blots were probed with the anti-myc mouse monoclonal (9E10), anti flag mouse monoclonal (F1804) and anti-tubulin alpha antibody (Rat monoclonal). The antibody concentration was 1:2500, 1:2500 and 1:1000 respectively. The secondary antibody used was goat anti-mouse for 9E10 and F1804 and goat anti-rat for anti-tubulin for 1:5000 dilution. The western blot was developed using TMB/H2O2 as a substrate.

### Live cell imaging using fluorescence microscopy

To analyze the defect in chromosomal segregation in cells, first cells were grown in SC medium supplement with 2% raffinose and induction was done for 4 h using 2% galactose as described earlier for protein extraction. CEN-V GFP dots were analyzed in cells that were fixed in 5% formaldehyde for 10 min, washed twice and stored in 0.1 M phosphate buffer pH 7.4. Before the microscopic analysis samples were fixed with 50% EtOH then wash with 0.1 M phosphate buffer and suspended in 1 μg/ml DAPI solution. Images were acquired using z-stack (at every 0.25 μm) multichannel image acquisition tool of Zeiss Axiovision software and Zeiss Axio Observer.Z1 microscope. Approximately 100 cells were counted per samples for statistical analysis.

### Frogging assay

To analyze the growth defect, the strains were first grown in synthetic complete medium devoid of uracil (raffinose was used as a carbon source) for overnight at 30 °C and 200 rpm shaking condition. Then secondary inoculation was done and the initial OD_600_ was maintained at ~ 0.2 and then grows till ~ 1.0 OD_600_. Equal cell from each sample were taken and different dilution was made from 1 to 10^− 5^ and spotting was done on the synthetic complete medium or dropout medium agar plates having galactose or dextrose as a carbon source separately.

## Supplementary information

**Additional file 1:** **Figure S1.** Characterization of pRS316-GAL-cDNA library (A) Image of some of the clones of pRS316-GAL-cDNA library digested with *Sal*I and *Not*I. Clone efficiency and average insert size were calculated. Out of these two values the number of colonies required for the screening was also calculated. (B) Transformation efficiency of the library by transforming yeast strain (CRY1) with 850 ng of cDNA library and transformants ware diluted for 1000 times and spread on the SC-ura plate.

## Data Availability

The data that support the findings of this study are available from the corresponding author upon reasonable request.

## Abbreviations

CCP: Centromeric cohesion protection
DAPI: 4′,6-diamidino-2-phenylindole
GFP: Green fluorescent protein
PP2A: Protein phosphatase 2A
PVDF: Polyvinylidene difluoride
SDS-PAGE: Sodium dodecyl sulfate polyacrylamide gel electrophoresis
SGO: Shugoshin
SKM: Sister Kinetochore monoorientation

## References

[1] Petronczki M, Siomos MF, Nasmyth K (2003). Un menage a quatre: the molecular biology of chromosome segregation in meiosis. Cell.

[2] Hauf S, Watanabe Y (2004). Kinetochore orientation in mitosis and meiosis. Cell.

[3] Cleveland DW, Mao Y, Sullivan KF (2003). Centromeres and kinetochores: from epigenetics to mitotic checkpoint signaling. Cell.

[4] Katis VL, Matos J, Mori S, Shirahige K, Zachariae W, Nasmyth K (2004). Spo13 facilitates monopolin recruitment to kinetochores and regulates maintenance of centromeric cohesion during yeast meiosis. Curr Biol.

[5] Watanabe Y (2005). Shugoshin: guardian spirit at the centromere. Curr Opin Cell Biol.

[6] Kiburz BM, Amon A, Marston AL (2008). Shugoshin promotes sister kinetochore biorientation in Saccharomyces cerevisiae. Mol Biol Cell.

[7] Mehta G, Anbalagan GK, Bharati AP, Gadre P, Ghosh SK (2018). An interplay between Shugoshin and Spo13 for centromeric cohesin protection and sister kinetochore mono-orientation during meiosis I in *Saccharomyces cerevisiae*. Curr Genet.

[8] Tóth A, Rabitsch KP, Gálová M, Schleiffer A, Buonomo SB, Nasmyth K (2000). Functional genomics identifies monopolin: a kinetochore protein required for segregation of homologs during meiosis I. Cell..

[9] Clyne RK, Katis VL, Jessop L, Benjamin KR, Herskowitz I, Lichten M, Nasmyth K (2003). Polo-like kinase Cdc5 promotes chiasmata formation and cosegregation of sister centromeres at meiosis I. Nat Cell Biol.

[10] Rabitsch KP, Petronczki M, Javerzat JP, Genier S, Chwalla B, Schleiffer A, Tanaka TU, Nasmyth K (2003). Kinetochore recruitment of two nucleolar proteins is required for homolog segregation in meiosis I. Dev Cell.

[11] Petronczki M, Matos J, Mori S, Gregan J, Bogdanova A, Schwickart M, Mechtler K, Shirahige K, Zachariae W, Nasmyth K (2006). Monopolar attachment of sister kinetochores at meiosis I requires casein kinase 1. Cell..

[12] Corbett KD, Yip CK, Ee LS, Walz T, Amon A, Harrison SC (2010). The monopolin complex crosslinks kinetochore components to regulate chromosome-microtubule attachments. Cell..

[13] Corbett KD, Harrison SC (2012). Molecular architecture of the yeast monopolin complex. Cell Rep.

[14] Gregan J, Riedel CG, Pidoux AL, Katou Y, Rumpf C, Schleiffer A, Kearsey SE, Shirahige K, Allshire RC, Nasmyth K (2007). The kinetochore proteins Pcs1 and Mde4 and heterochromatin are required to prevent merotelic orientation. Curr Biol.

[15] Riedel CG, Katis VL, Katou Y, Mori S, Itoh T, Helmhart W, Gálová M, Petronczki M, Gregan J, Cetin B, Mudrak I (2006). Protein phosphatase 2A protects centromeric sister chromatid cohesion during meiosis I. Nature.

[16] Kitajima TS, Sakuno T, Ishiguro KI, Iemura SI, Natsume T, Kawashima SA, Watanabe Y (2006). Shugoshin collaborates with protein phosphatase 2A to protect cohesin. Nature..

[17] Kitajima TS, Kawashima SA, Watanabe Y (2004). The conserved kinetochore protein shugoshin protects centromeric cohesion during meiosis. Nature..

[18] Marston AL, Tham WH, Shah H, Amon A (2004). A genome-wide screen identifies genes required for centromeric cohesion. Science.

[19] Rabitsch KP, Gregan J, Schleiffer A, Javerzat JP, Eisenhaber F, Nasmyth K (2004). Two fission yeast homologs of Drosophila Mei-S332 are required for chromosome segregation during meiosis I and II. Curr Biol.

[20] Shonn MA, McCarroll R, Murray AW (2002). Spo13 protects meiotic cohesin at centromeres in meiosis I. Genes Dev.

[21] Lee BH, Kiburz BM, Amon A (2004). Spo13 maintains centromeric cohesion and kinetochore coorientation during meiosis I. Curr Biol.

[22] Dudas A, Ahmad S, Gregan J (2011). Sgo1 is required for co-segregation of sister chromatids during achiasmate meiosis I. Cell Cycle.

[23] Hirose Y, Suzuki R, Ohba T, Hinohara Y, Matsuhara H, Yoshida M, Itabashi Y, Murakami H, Yamamoto A (2011). Chiasmata promote monopolar attachment of sister chromatids and their co-segregation toward the proper pole during meiosis I. PLoS Genet.

[24] Kagami A, Sakuno T, Yamagishi Y, Ishiguro T, Tsukahara T, Shirahige K, Tanaka K, Watanabe Y (2011). Acetylation regulates monopolar attachment at multiple levels during meiosis I in fission yeast. EMBO Rep.

[25] Yamagishi Y, Sakuno T, Goto Y, Watanabe Y (2014). Kinetochore composition and its function: lessons from yeasts. FEMS Microbiol Rev.

[26] Monje-Casas F, Prabhu VR, Lee BH, Boselli M, Amon A (2007). Kinetochore orientation during meiosis is controlled by Aurora B and the monopolin complex. Cell..

[27] Varela E, Schlecht U, Moina A, Fackenthal JD, Washburn BK, Niederhauser-Wiederkehr C, Tsai-Pflugfelder M, Primig M, Gasser SM, Esposito RE (2010). Mitotic expression of Spo13 alters M-phase progression and nucleolar localization of Cdc14 in budding yeast. Genetics..

[28] Charles JF, Jaspersen SL, Tinker-Kulberg RL, Hwang L, Szidon A, Morgan DO (1998). The polo-related kinase Cdc5 activates and is destroyed by the mitotic cyclin destruction machinery in S. cerevisiae. Curr Biol.

[29] Liu H, Krizek J, Bretscher A (1992). Construction of a GAL1-regulated yeast cDNA expression library and its application to the identification of genes whose overexpression causes lethality in yeast. Genetics..

[30] Kim J, Ishiguro KI, Nambu A, Akiyoshi B, Yokobayashi S, Kagami A, Ishiguro T, Pendas AM, Takeda N, Sakakibara Y, Kitajima TS (2015). Meikin is a conserved regulator of meiosis-I-specific kinetochore function. Nature.

[31] Mehta GD, Agarwal M, Ghosh SK (2014). Functional characterization of kinetochore protein, Ctf 19 in meiosis I: an implication of differential impact of Ctf 19 on the assembly of mitotic and meiotic kinetochores in *Saccharomyces cerevisiae*. Mol Microbiol.

[32] Zhang T, Lei J, Yang H, Xu K, Wang R, Zhang Z (2011). An improved method for whole protein extraction from yeast *Saccharomyces cerevisiae*. Yeast.

