# Supplementary material for: Construction of strains to identify novel factors for regulation of centromeric cohesion protection (CCP) and sister kinetochore mono-orientation (SKM)
Source: BMC Mol Cell Biol. 2019 Oct 22;20:44. doi: 10.1186/s12860-019-0231-2 (PMC6806570; doi:10.1186/s12860-019-0231-2)
Supplement: Supplementary file 1 — Additional file 1: Figure S1. Characterization of pRS316-GAL-cDNA library (A) Image of some of the clones of pRS316-GAL-cDNA library digested with SalI and NotI. Clone efficiency and average insert size were calculated. Out of these two values the number of colonies required for the screening was also calculated. (B) Transformation efficiency of the library by transforming yeast strain (CRY1) with 850 ng of cDNA library and transformants ware diluted for 1000 times and spread on the SC-ura plate. [file 12860_2019_231_MOESM1_ESM.docx]

**
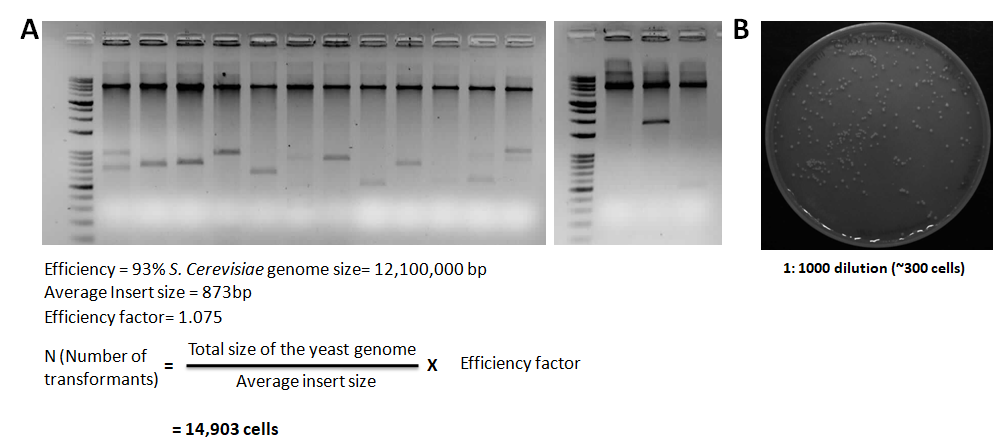
**

**Figure S1. Characterization of pRS316-GAL-cDNA library**

(A) Image of some of the clones of pRS316-GAL-cDNA library digested with *Sal*I and *Not*I. Clone efficiency and average insert size were calculated. Out of these two values the number of colonies required for the screening was also calculated. (B) Transformation efficiency of the library by transforming yeast strain (CRY1) with 850 ng of cDNA library and transformants ware diluted for 1000 times and spread on the SC-ura plate.
